# Supplementary material for: Poly(vinylidene fluoride) Composite Nanofibers Containing Polyhedral Oligomeric Silsesquioxane–Epigallocatechin Gallate Conjugate for Bone Tissue Regeneration
Source: Nanomaterials (Basel). 2019 Feb 1;9(2):184. doi: 10.3390/nano9020184 (PMC6409983; doi:10.3390/nano9020184)
Supplement: Supplementary file 1 [file nanomaterials-09-00184-s001.pdf]

Supporting Information

Poly(vinylidene fluoride) Composite Nanofibers Containing Polyhedral Oligomeric Silsesquioxane–Epigallocatechin Gallate Conjugate for Bone Tissue Regeneration

Hyo-Geun Jeong [1], Yoon-Soo Han [2], Kyung-Hye Jung [2] and Young-Jin Kim [1,*]

[1] Department of Biomedical Engineering, Daegu Catholic University, Gyeongsan 38430, Republic of Korea; jhg2833@empal.com (H.-G.J.); yjkim@cu.ac.kr (Y.-J.K.)

[2] Department of Advanced Materials and Chemical Engineering, Daegu Catholic University, Gyeongsan 38430, Republic of Korea; yshancu@cu.ac.kr (Y.-S,H.); khjung@cu.ac.kr (K.-H.J.)

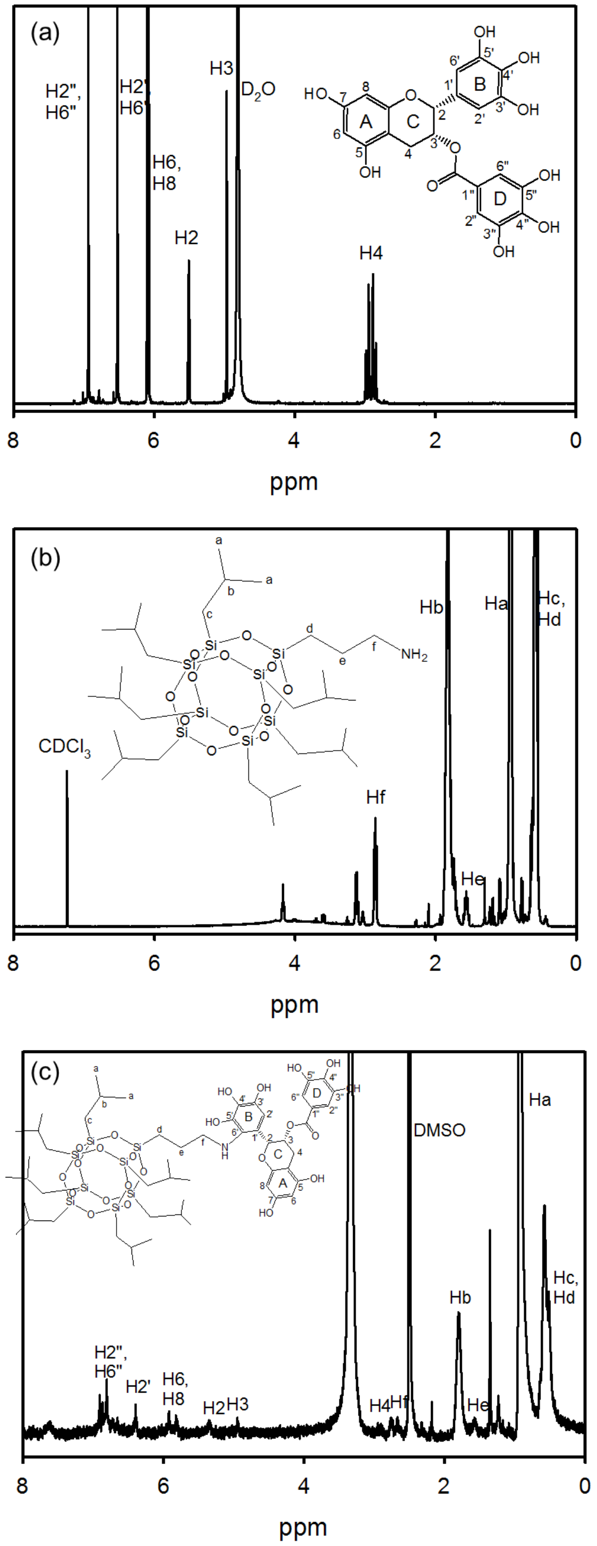

Figure S1. ^{1}H NMR spectra of (a) EGCG, (b) API-POSS, and (c) POSS-EGCG conjugate.

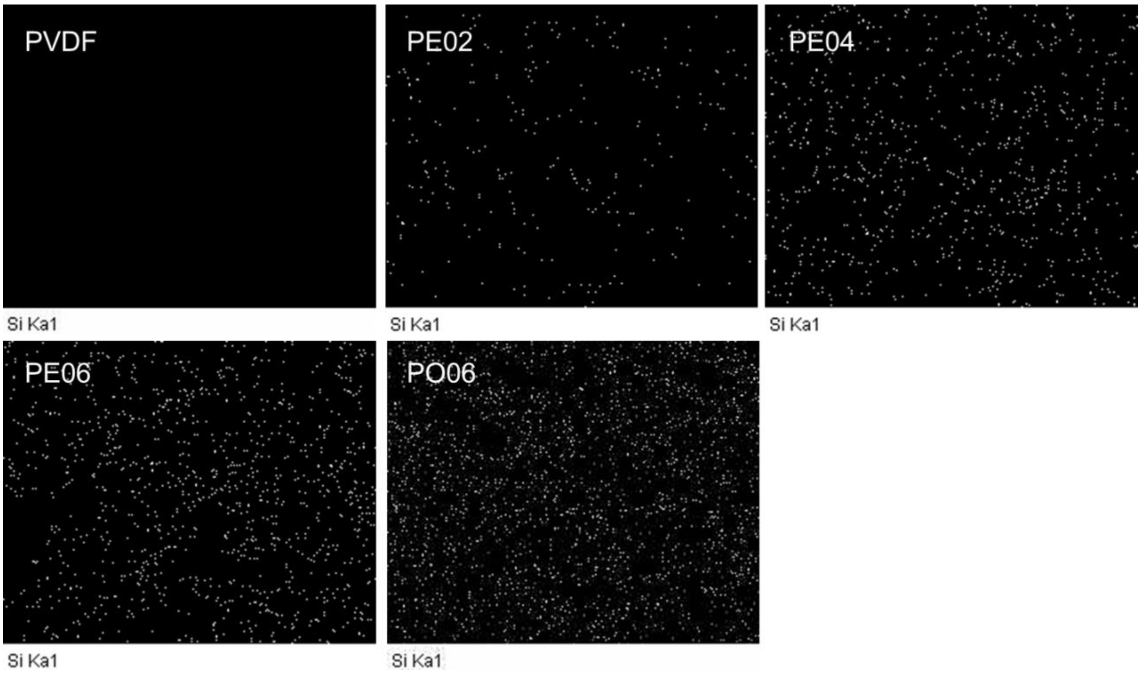

Figure S2. EDS Si-mapping images of the PVDF composite nanofibers.
